# Atrial Septal Defects Presenting Initially in Adulthood: Patterns of Clinical Presentation in Enugu, South-East Nigeria

**DOI:** 10.1155/2011/251913

**Published:** 2011-05-14

**Authors:** E. C. Ejim, B. C. Anisiuba, S. O. Ike, I. O. Essien

**Affiliations:** ^1^Department of Medicine, University of Nigeria Teaching Hospital, P.M.B. 01129, Ituku-Ozalla, Enugu, Nigeria; ^2^Department of Medicine, University of Uyo Teaching Hospital, PMB 1136, Uyo, Nigeria

## Abstract

This paper aimed to evaluate the patterns of clinical presentation of adults with atrial septal defects (ASDs) who were diagnosed from transthoracic echocardiographic examination at the echocardiographic laboratory of the University of Nigeria Teaching Hospital Ituku-Ozalla, Enugu, Nigeria, from February 2002 to June 2010. 2251 new echocardiogram scans, with additional 373 repeat scans, were done within the period. 32 adults had ASDs (1.3%), made up of 9 males and 23 females. Secundum ASD constituted 75% while dyspnoea on exertion was the commonest symptom. Congestive cardiac failure was the clinical syndrome most commonly encountered, and most patients presented in the third decade. This paper demonstrated that ASDs are common congenital heart diseases in adult Nigerians, and that they are important causes of congestive heart failure. All adults with congestive heart failure must be referred for echocardiography for early identification of causes like ASDs, which are often forgotten, before the development of irreversible changes in the lungs.

## 1. Introduction

In the developed countries, more than 95% of children with congenital heart defects reach adulthood currently [1]. In Europe, the number of adults with congenital heart disease is estimated to be at least 1.2 million, and despite major developments in diagnostic methods and treatment of congenital heart disease, cure is rarely achieved [2, 3].

Atrial septal defect (ASD) is one of the most common congenital heart defects in adults [4, 5]. ASD patients can present at any age, and females constitute 65–75% of patients with the secundum type while the gender distribution is equal for sinus venosus and ostium primum types [6, 7]. Exercise intolerance is the most common initial presenting symptom while palpitation, features of decompensated heart failure, paradoxical embolus, or cyanosis may also be the presenting feature [6].

The authors are not aware of any studies reporting prevalence of atrial septal defects in adults or the pattern of presentation of these patients in sub-Saharan African.

In low-income settings like in most sub-Saharan African countries, echocardiography is not readily available and/or affordable. It then becomes important that clinicians recognize the clinical features of these conditions and make prompt referrals before the development of irreversible changes in the lungs and heart.

## 2. Materials and Methods

The University of Nigeria Teaching Hospital (UNTH) Enugu, Enugu State, is a 700-bed tertiary hospital in South-East Nigeria. Within the period under review, it provided echocardiographic services for about 35 million Nigerians in the South East region. Although many new centres currently offer echo services, the UNTH remains a referral centre for other tertiary institutions in South East Nigeria. The hospital is designated the cardiothoracic centre of excellence in Nigeria and was the only centre in Nigeria providing cardiac surgery routinely up till the year 2005.

Congenital heart disease, hypertension, and rheumatic heart disease were the commonest indications for echocardiography at the UNTH [8].

Most patients were referred for echo from the cardiothoracic units of the hospital (adult cardiology, pediatric cardiology, cardiac surgery) while a few were referred from other units in the hospital and other hospitals within Nigeria.

Consecutive echocardiogram reports of 2251 patients done over a period of 8 years (from February 2002 to June 2010) were retrospectively reviewed out of a total of 2624 scans. Repeat scans were excluded from the count, hence the difference.

All adults (aged 18 years and above) who had isolated atrial septal defects on transthoracic echocardiography were included in the study. The case records of these patients were retrieved, and data obtained included age at presentation, gender, and common clinical features as well as presence or absence of central cyanosis. Patients who were diagnosed in childhood, but who did not have corrective surgery or who were lost to followup were excluded from the study.

The clinical diagnosis on the echo request form was taken as the working diagnosis, and the data available on these forms were used for referred patients whose case notes could not be traced to the referring hospital.

Echocardiography was done with a Hewlett Packard SONOS 2000 echocardiographic machine equipped with a 3.7 MHz transducer for adults and 5.5 MHz transducer for children, a video recorder, and printout processor. The machine has M-mode, two-dimensional (2-D), and Doppler facilities. Performance and reading of echocardiograms were done by three cardiologists. The video records of all patients with ASD were reviewed together by the three cardiologists, and only those cases where the cardiologists agreed were recruited for the study.

All measurements were taken according to the recommendation of the American Society of Echocardiography [9]. 

Informed consent was obtained from the patients verbally before performing the examinations. Results were analyzed with a computer using SPSS version 15.

## 3. Results

A total of two thousand five hundred and twenty-one new echocardiographic examinations were done over the period of review.

Thirty-two adults had atrial septal defect representing 1.3% of all the new echocardiogram done over this period. The age range of these patients was from 18–80 years with a mean of 32.8 ± 14.3. There were 9 males (28%) and 23 females (72%) with a male-to-female ratio of 1 : 2.5. The mean age of the males was 39.0 ± 4.2 while the mean age of the females was 35.5 ± 13.9.

Secundum ASD was seen in 24 patients (75%), primum ASD in 4 patients (12.5%), and sinus venosus ASD in 4 patients (12.5%).

The commonest clinical symptoms were shortness of breath on exertion (25%), palpitation (21%), and cough (21%) (Table 1).

Cyanosis was present in 12.5% of the patients while pulmonary hypertension on echocardiography was seen on 21% of the patients.

The commonest clinical diagnoses were congestive cardiac failure, rheumatic heart disease, and atrial septal defect (Table 2). 

We also observed that most of the patients in our study presented in the 3rd decade of life (Table 3).

## 4. Discussion

This study has shown that atrial septal defect is a relatively common congenital heart disease in adults, and also an important cause of congestive cardiac failure, presenting as the syndrome of heart failure in 21% of the patients. 

The decline in the incidence and prevalence of rheumatic fever has resulted in a reduction in the prevalence of rheumatic heart disease. 

Rheumatic heart disease is a very important cause of heart failure in young adults in sub-Saharan Africa [10], and it is often confused with complicated atrial septal defect as a cause of heart failure. The importance of atrial septal defect as a cause of heart failure will become more obvious as the results of various government programmes and policies to eradicate infectious and communicable diseases become manifest in the near future.

Clinical diagnosis of atrial septal defect was correct in 15% of the cases representing 3 out of 20 patients which is very unsatisfactory. This brings to fore the need for echocardiographic services to be made available and affordable at all tertiary health facilities in this part of the world.

All cases of heart failure must be referred for echocardiography.

However, in the event of nonavailability/affordability of these facilities which is the case in most sub-Saharan African countries, teachers of cardiology should still impart to the medical students the skills for eliciting the clinical signs of important cardiac conditions including congenital heart diseases in adults. The usefulness of these basic skills cannot be overemphasized in our environment until such a time when the personnel and equipments for such investigations are available and affordable to all those who need them.

ASD was commoner in females in our study as reported in many other studies, and secundum ASD was the commonest type as reported by other workers [5, 6].

Dyspnoea or exertion was the commonest symptoms among the patients we studied, and this is similar to reports from other workers [5, 6, 11].

The asymptomatic nature of atrial septal defect, and the failure to see the need for regular medical examination in the absence of ill health in this part of the world makes our patients present with complications. Most patients in this study (about 80%) presented between the ages of 21 and 50 years, and these individuals constitute a greater majority of the workforce of the nation. The impact of the morbidity of ASD on the individual, as well as on his nation is definitely enormous. 

This state of affairs will continue until the level of poverty in these countries is reduced to a point where most people will be able to include health matters in their annual budget.

## 5. Conclusion

Atrial septal defect is a common congenital anomaly in adults living in Nigeria, and it is often forgotten as a cause of congestive heart failure.

Early recognition may help those patients who have the wherewithal to go for closure of the defect and forestall the complications which are often burdensome.

## Figures and Tables

**Table 1:** Common symptoms at presentation.

Symptoms	Frequency (%)
Dyspnoea on exertion	26
Cough	21
Palpitation	16
Leg swelling	10.5
Chest pain	10.5
Paroxysmal nocturnal dyspnoea	10.5
Abdominal distension	5.3

**Table 2:** Common clinical diagnosis.

Diagnosis	Frequency (%)
CCF from valvular heart disease	21
Rheumatic heart disease	18
Atrial septal defect	15
Congenital heart disease	9
Ventricular septal defect	9
Arrhythmia	9
Cardiomyopathy	6
Hypertensive heart disease	6
Complete heart block	3
Others	4

**Table 3:** Distribution of ASD according to age of presentation.

Age (yrs)	Frequency (%)
18–20	9.4
21–30	37.5
31–40	21.8
41–50	18.3
51–60	6.2
61–70	3.1
71–80	3.1
